# Stress-Induced Neuroprotective Effects of Epiregulin and Amphiregulin

**DOI:** 10.1371/journal.pone.0118280

**Published:** 2015-02-12

**Authors:** Libin Zhan, Luping Zheng, Toru Hosoi, Yasunobu Okuma, Yasuyuki Nomura

**Affiliations:** 1 Department of Traditional Chinese Medicine, The Second Affiliated Hospital, Dalian Medical University, Dalian, China; 2 Department of Pharmacology, Graduate School of Pharmaceutical Sciences, Hokkaido University, Sapporo, Japan; 3 College (Institute) of Integrative Medicine, Dalian Medical University, Dalian, China; 4 Department of Pharmacotherapy, Graduate School of Biomedical and Health Sciences, Hiroshima University, Hiroshima, Japan; 5 Department of Pharmacology, Faculty of Pharmaceutical Sciences, Chiba Institute of Sciences, Choshi, Japan; 6 Department of Pharmacology, Kurume University School of Medicine, Kurume, Fukuoka, Japan; Massachusetts General Hospital/Harvard Medical School, UNITED STATES

## Abstract

Members of the epidermal growth factor family play important roles in the regulation of cell growth, proliferation, and survival. However, the specific roles of each epidermal growth factor family member with respect to brain injury are not well understood. Gene chip assay screens have revealed drastic increases in the expression of the epidermal growth factor family members amphiregulin and epiregulin following lipopolysaccharide stimulation, which activates an immune response. Both immune activity and endoplasmic reticulum stress are activated during cerebral ischemia. We found that the expression levels of amphiregulin and epiregulin were significantly increased under conditions of cerebral ischemia. Because endoplasmic reticulum stress increased the expression of amphiregulin and epiregulin in glial cells, endoplasmic reticulum stress may be a key mediatory factor of pathophysiological activity. Recombinant epiregulin and amphiregulin proteins effectively inhibited endoplasmic reticulum stress and the subsequent induction of neuronal cell death. Therefore, the upregulation of the epidermal growth factor family members epiregulin and amphiregulin may play a critical role in preventing endoplasmic reticulum stress-induced cell death, thus providing a potential therapy for brain injury.

## Introduction

Members of the epidermal growth factor (EGF) family are known to stimulate the proliferation, differentiation, and survival of numerous cell types. Most recently, the roles of EGF family members in the brain and central nervous system (CNS) have attracted considerable attention. In particular, the roles of these proteins in neuroprotection have been investigated. EGF has been shown to protect against neurotoxicity or cerebral ischemia-induced dopaminergic neuronal death [1, 2]. Heparin-binding EGF-like growth factor (HB-EGF) has been proven to act as an endogenous neuroprotective agent following brain injury [3, 4]. Transforming growth factor alpha (TGF-α) exerts neurotrophic effects and therefore guarantees neuronal survival and inhibits apoptosis [5]. Recent studies have suggested that betacellulin (BTC) could stimulate the growth of neural stem cells (NSC) and precursor cells in the brain and also increase neurogenesis [6, 7].

Previous studies of the EGF family members amphiregulin (AR) and epiregulin (EPR) have mainly focused on their roles in cell proliferation and tumorigenesis within the mammary gland, ovary, and other organs as well as glioblastoma. Falk and Frisén [8] reported the expression of AR in the choroid plexus of the ventricular system and the hippocampus of the adult brain and noted that AR may participate in the regulation of NSC proliferation and neurogenesis in those areas. AR expression was found to be upregulated following transection of the sciatic nerve, and the addition of AR was found to stimulate axonal outgrowth from the mouse dorsal root ganglia (DRG); moreover, AR acts as an autocrine survival factor for adult sensory neurons [9, 10]. In a similar study, AR mRNA expression was induced in the DRG of TGF-α knockout mice after sciatic nerve injury [11]. These results suggest that AR is important for nerve survival and regeneration. Furthermore, subcutaneous EPR administration has been shown to increase the phosphorylation of the brain EGF receptor ErbB1 in mouse neonates, suggesting the potential effect of EPR on brain development [12]. A recent study demonstrated that EPR induces human neuroblastoma cell differentiation via the ERK1/2 signaling pathway [13]. Although the above-described studies imply a potential neuroprotective effect, studies involving a direct approach and functional studies of AR and EPR in the injured brain and nerve cells are limited.

In this paper, we induced lipopolysaccharide (LPS)-mediated inflammatory reactions, cerebral ischemia, hypoxia, and endoplasmic reticulum stress (ER Stress) in the brain, primary glial cells, and neuron and demonstrated that these challenges and stress conditions directly resulted in brain and nerve cell damage, including apoptosis. We then investigated the expression patterns of EGF family members, particularly EPR and AR, and their roles in neuroprotection.

## Materials and Methods

### Cell culture

Primary glial cells were prepared from the whole brains of neonatal (24 h) C57BL/6 mice as described previously [14]. The cells were allowed to grow to confluence in Dulbecco’s modified Eagle’s medium with 10% (v/v) fetal calf serum, 100 U/ml of penicillin G, and 100 μg/ml of streptomycin (Invitrogen, Carlsbad, CA, USA). Mouse neuroblastoma Neuro2a cells which were purchased from American Type Culture Collection (ATCC) were maintained in modified Eagle’s medium supplemented with 10% (v/v) heat-inactivated fetal calf serum. All cultured cells were maintained at 37°C in 5% CO_2_/95% air.

### Gene chip and quantitative assays

To investigate the potential induction of inflammatory cytokines following LPS challenge, we screened several potential molecules, including 2 typical pro-inflammatory cytokines and 6 EGF family members, using a gene chip probe array. Primary cultured glial cells were exposed to LPS (10 μg/mL) for 4 h, and total RNA was isolated using TRI REAGENTi (Sigma-Aldrich Corporation, St. Louis, MO, USA). Two independent sets of these glial cells were pooled (25 μg of total RNA/sample) and analyzed using the GeneChip Mouse Expression Set 430A (Affymetrix Inc., San Diego, CA, USA). We entrusted the analysis of our array to GeneticLab Co., Ltd. (Sapporo, Japan).

### LPS-induced inflammatory reaction

LPS exposure experiments were conducted to evaluate the time- and dose-dependent responses and investigate the expression of EGF family member mRNA. Primary cultured glial cells were treated with 1 μg/ml of LPS for 0, 1, 2, 4, 8, and 24 h or with gradually increasing LPS doses of 0.01, 0.1, 1, and 10 μg/ml for 4 h. We then collected the cultured cells from the different treatment regimes and isolated the total RNA for real-time polymerase chain reaction (RT-PCR) analysis.

### Hypoxia induction in mouse primary glial cells

Sub-confluent primary cultured glial cells were exposed to a mixture of CO_2_ (5%) and N_2_ in a humidified incubator (ANX-1, HIRASAWA, Tokyo, Japan) at 37°C within a sealed, anaerobic, gloved cabinet containing a catalyst to scavenge free oxygen molecules. The oxygen pressures in the chamber and medium were measured using an oxygen analyzer (Teledyne Technologies, Thousand Oaks, CA, USA) and a blood gas analyzer (ABL-2; Radiometer, Copenhagen, Denmark), respectively. Throughout the incubation period, the oxygen levels in the cabinet were measured using a monitor sensitive to oxygen concentrations <10 ppm. The oxygen pressures in the cultured medium at 30 min, 1 h, 6 h, and 48 h fell to 4.3, 3.0, 2.2, and 2.0 ± 0.2%, respectively, after transfer into the hypoxic chamber. The medium was then changed and the cells were cultured under normal conditions for the indicated time periods.

### Murine hypoxic/ischemia model

The mouse hypoxia/ischemia assay was performed as described previously [15]. In brief, 6-week-old C57BL/6 mice purchased from Charles River (Yokohama, Japan) were anesthetized with halothane (2% in 70% N_2_O:30% O_2_) and in each, the right carotid artery was isolated and double ligated with 4–0 surgical thread. The incision was then sutured, and the animals were allowed to recover with free access to food and water for 3 h. To induce hypoxia, each animal was placed in a 500-ml glass jar partially submerged in a temperature-controlled water bath and exposed to a humidified gas mixture of 6% O_2_/balance N_2_ for 30-min intervals. The animals were then allowed to recover in room air for 30 min before returning to their cages with free access to food and water. Animals that underwent surgical separation but not ligation of the right common carotid artery and were subjected to hypoxia were used as sham-operated controls. All animal experiments were carried out in accordance with the National Institutes of Health Guide for the Care and Use of Laboratory Animals and were approved by the animal care and use committee of Hokkaido University.

### ER stress and lactate dehydrogenase (LDH) leakage assay

Primary glial cells were cultured as described above until unilaminar cells covered 70% of the flask. The cells were then stimulated with 3 μg/ml of tunicamycin (Tm) for 0, 3, 6, 12, 24, and 48 h, after which total RNA was extracted for RT-PCR. To investigate the neuroprotective effects of EPR and AR, Neuro2a cells were pretreated with recombinant EPR (0, 0.01, 0.03, 0.1, and 1 nM) and AR (0, 0.001, 0.01, 0.03, 0.1, and 1 nM) proteins diluted in 10% serum for 1 h. Next, 1 μg/ml of Tm was added to the recombinant protein-treated cells, followed by a 48-h incubation. The viability of Neuro2a cells following Tm stimulation was estimated by the LDH leakage assay and a cytotoxicity detection kit (Roche Molecular Biochemicals, Indianapolis, IN, USA) according to the manufacturer’s instructions. The LDH activity was measured as the optimal density at 492 nm, and LDH leakage (%) was defined as the ratio of LDH activity in the culture medium to the total activity.

### RT-PCR analysis

RT-PCR was performed as described previously [16]. Specifically, cDNA was synthesized from total RNA via reverse transcription with 100 U of Superscript III Reverse Transcriptase and Oligo (dt)_12–18_ (Invitrogen, Carlsbad, CA, USA) in a 20-μl reaction mixture containing Superscript buffer, 1 mM dNTP mix, 10 mM dithiothreitol, and 40 U of RNase inhibitor. For PCR amplification, 1.2 μL of cDNA was added to 12 μL of a reaction mix containing 0.2 μM of each primer, 0.2 μM of dNTP mix, 0.6 U of Taq polymerase, and reaction buffer. PCR was performed in a DNA Thermal Cycler (GeneAmp PCR System 9700; Applied Biosystems, Carlsbad, CA, USA). The primers for each PCR are presented in S1 Table.

### Western blot analysis

Cultured Neuro2a cells were collected, washed with phosphate-buffered saline (PBS), and lysed in a lysis buffer. The protein concentrations were measured using the Bio-Rad Protein Assay kit (Bio-Rad Laboratories, Hercules, CA, USA). The total protein extracts were then fractionated by SDS-PAGE and transferred to nitrocellulose membranes at 4°C. The membranes were subsequently incubated in blocking buffer at room temperature followed by an overnight incubation with a primary antibody at 4°C. The membranes were then incubated with a horseradish peroxidase (HRP)-labeled secondary antibody diluted 1:2000 in Tris-buffered saline + Tween-20 with 5% milk for 1 h at room temperature. HRP-labeled antibodies bound to the membranes were detected by chemiluminescence.

### Statistical Analysis

Statistical analyses were performed using Excel (Microsoft Corporation, Redmond, WA, USA). A 1-way analysis of variance was used to determine the differences between the groups (significance level, P < 0.05; t-test) in the software package SPSS 16.0 (SPSS, Inc., Chicago, IL, USA). The data are presented as mean ± standard errors of the mean, and each experiment was performed in triplicates.

## Results

### The EPR and AR expression patterns in response to LPS challenge

To evaluate the roles of EGF family members during inflammatory reactions, we measured the expression levels of the pro-inflammatory cytokines inducible nitric oxide synthase (iNOS) and cyclooxygenase-2 (Cox-2) in primary cultured glial cells using a Gene Chip Probe Array. The results indicated that the expression of both investigated pro-inflammatory cytokines was induced significantly following LPS stimulation (Table 1). Under these conditions, we found that the expression of both EPR and AR was significantly induced relative to that of other EGF family members (Table 2), suggesting that EPR and AR may play important roles during inflammatory reactions in the brain and nerve cells.

**Table 1:** Fold changes in pro-inflammatory cytokines expression following LPS stimulation.

Pro-inflammatory cytokines	Fold change
iNOS	56
Cox-2	52

**Table 2:** Fold changes in EGF family member expression following LPS stimulation.

EGF family members	Fold change
Amphiregulin	32
Epiregulin	11.3
EGF	1.1
Betacellulin	3.2
HB-EGF	1.4
TGF-a	0.9

**Fig 1:**
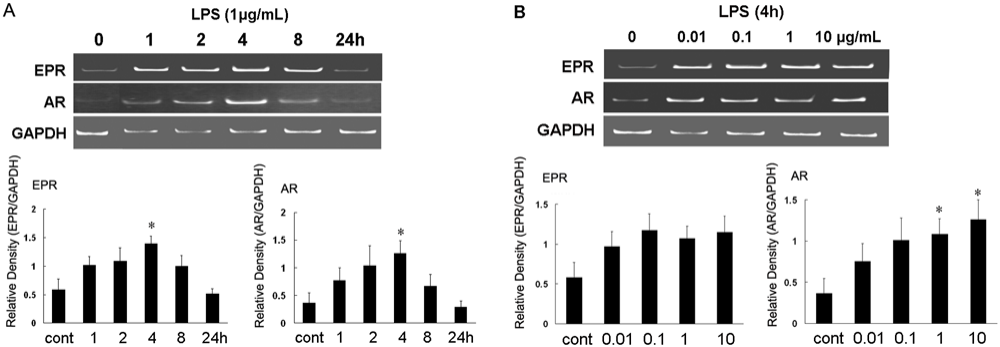
Induction of epiregulin (EPR) and amphiregulin (AR) mRNA expression in primary glial cells following lipopolysaccharide (LPS) treatment (time course and dose course). (A) Total RNA was isolated from cells exposed to LPS (1 μg/ml) for the indicated time periods and subjected to RT-PCR. (B) Total RNA was isolated from cells exposed to LPS (4 h) at the indicated concentrations and subjected to RT-PCR. Data are presented as the means ± standard errors from 3 separate experiments. *p < 0.05 compared with the control.

A time- and dose-dependent LPS stimulation experiment was then performed in primary glial cells to further investigate the EPR and AR expression levels. Following LPS stimulation, the expression patterns of AR, EPR, EGF, BTC, HB-EGF, and TGF-α were investigated by RT-PCR. All EGF members demonstrated similar expression patterns. Specifically, after LPS stimulation, the expression levels increased gradually before reaching a peak level at 4 h and then decreased to the minimum expression level at 24 h. Significant increases in EPR and AR mRNA levels were observed at 4 h when compared with the control levels (Fig. 1A). We observed a significant increase in AR expression when using either 1 or 10 μg/mL of LPS to stimulate primary glial cells (Fig. 1B). The expression levels of other EGF family members are presented in S1 Fig.

### Upregulation of EPR and AR following ischemia and hypoxia

**Fig 2:**
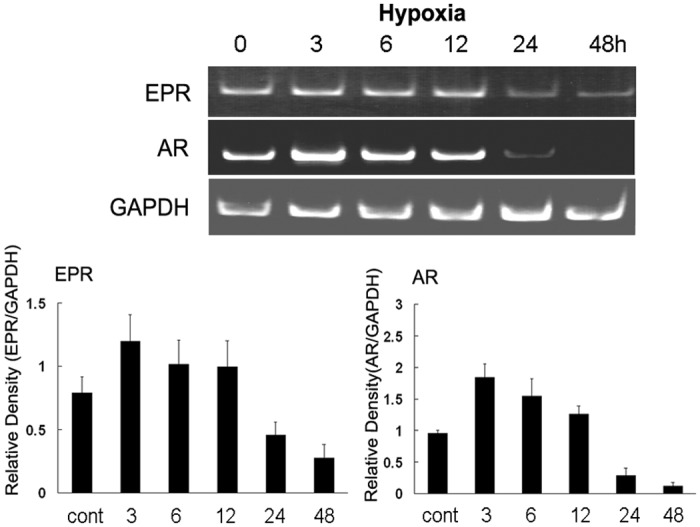
Induction of epiregulin (EPR) and amphiregulin (AR) mRNA expression in primary glial cells under hypoxic conditions. Total RNA was isolated from cells exposed to hypoxic conditions for the indicated time periods and subjected to RT-PCR. Data are presented as the means ± standard errors from 3 separate experiments. *p < 0.05 compared with the control.

**Fig 3:**
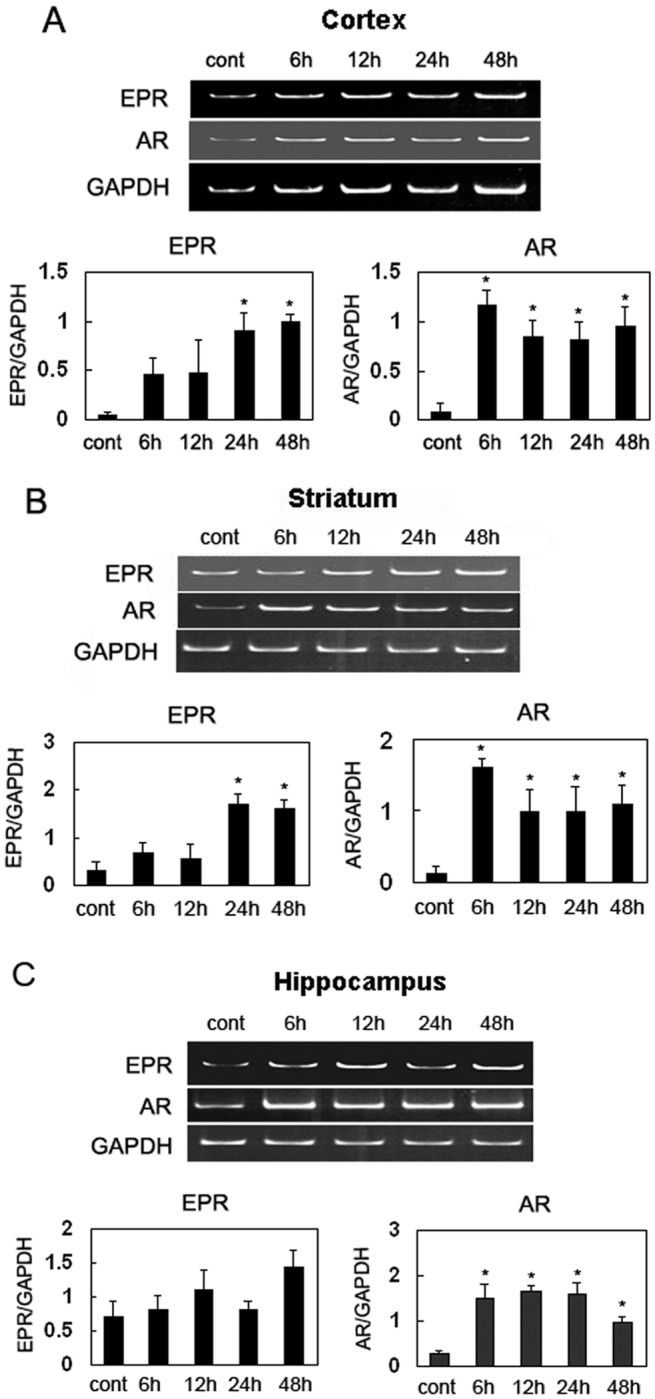
Time course analysis of epiregulin (EPR) and amphiregulin (AR) mRNA expression in the ischemic cerebral cortex. (A) The injured cortex, (B) striatum, and (C) hippocampus were obtained at various time points (control, 6, 12, 24, and 48 h) following a 30-min hypoxia/ischemia exposure; total RNA was extracted and subjected to RT-PCR. Data are presented as means ± standard errors. Control samples were obtained at 3 h after the sham operation (cont). *p < 0.05 compared with the sham-operated mice.

We cultured primary glial cells under hypoxic conditions *in vitro* and used RT-PCR to reveal that the levels of all EGF family members after 3 h in culture increased above the control levels before gradually decreasing. We also identified significant increases in AR expression after 3 h and 6 h in culture. In addition, the HB-EGF levels were also elevated, with a peak value appearing at 3 h (Fig. 2 and S2 Fig.). We further investigated the expression patterns of AR, EPR, EGF, BTC, HB-EGF, and TGF-α in the cortex, striatum, and hippocampus via RT-PCR following an *in vivo* ischemia assay. In the cortex, we observed significant differences in the levels of EPR mRNA at 24 h when compared with the control levels. This increase was found to persist until 48 h. The high expression levels of EPR and BTC were maintained throughout the analysis period, and significant differences in the control levels were observed between 6 h and 48 h (Fig. 3A). We did not observe any significant differences in the expression levels of other EGF family members when compared with the control levels (S3A Fig.). Similar EGF family member expression patterns were observed in the striatum (Fig. 3B and S3B Fig.). In the hippocampus, only AR expression increased significantly, and this increase persisted until 48 h after ischemia (Fig. 3C and S3C Fig.).

### EPR and AR play important roles in ER Stress in glial and Neuro2a cells

**Fig 4:**
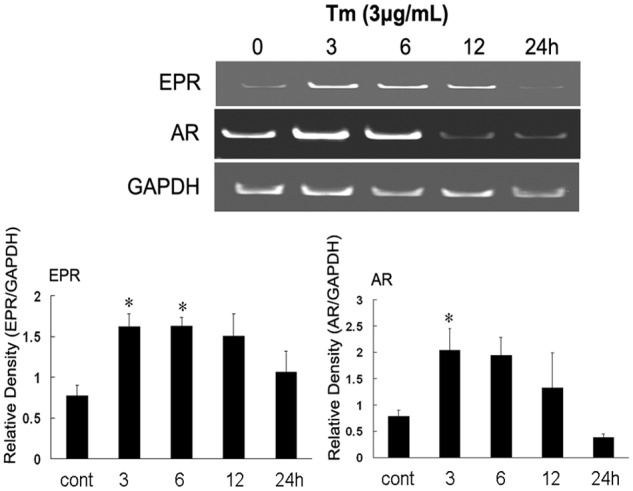
Induction of epiregulin (EPR) and amphiregulin (AR) mRNA expression in primary glial cells following Tm treatment. Total RNA was isolated from cells exposed to Tm (3 μg/ml) for the indicated periods and subjected to RT-PCR. Data are presented as the means ± standard errors from 3 separate experiments. *p < 0.05 compared with the control.

**Fig 5:**
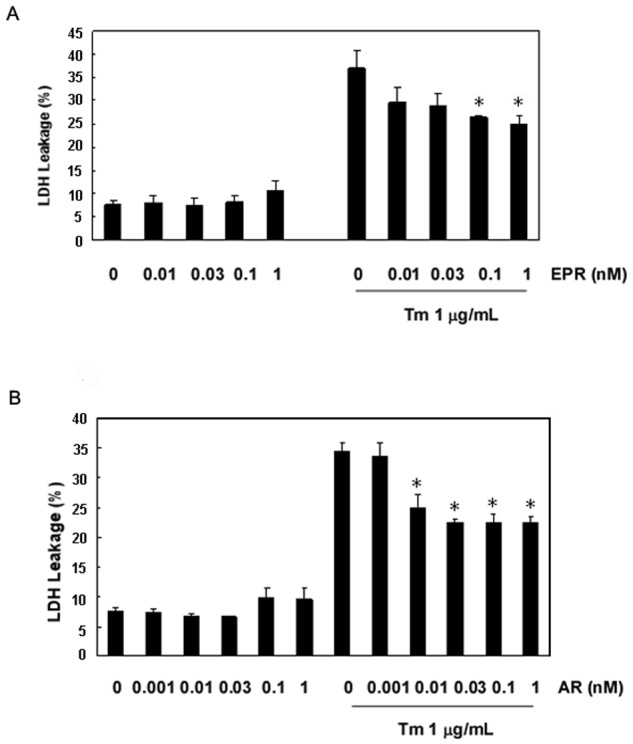
Epiregulin (EPR) and amphiregulin (AR) inhibited cell death in Neuro2a cells. The cells were pretreated with EPR (0.01, 0.03, 0.1, and 1 nM) and AR (0.001, 0.01, 0.03, 0.1, and 1 nM) for 1 h and subsequently treated according to the indicated conditions. (A) EPR reduced the release of lactate dehydrogenase (LDH) at 48 h after Tm (1 μg/mL) treatment. (B) AR reduced the release of LDH at 48 h after Tm (1 μg/mL) treatment. The LDH released into the medium was expressed as a percentage of the control value. The data represent the average of 4 independent experiments for each sample. *p < 0.05.

**Fig 6:**
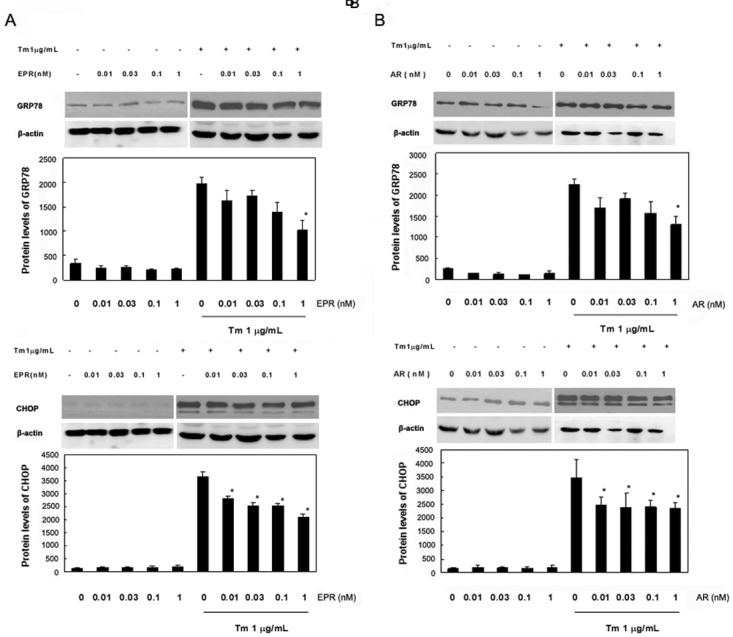
Epiregulin (EPR) and amphiregulin (AR) inhibited endoplasmic reticulum stress in Neuro2a cells. Neuro2a cells were pre-incubated with (A) EPR or (B) AR for 1 h and then treated with tunicamycin (Tm) for 24 h. GRP78 and CHOP expression was detected via western blotting. Protein quantification is expressed as the mean ± standard error of 3 independent experiments. *p < 0.05 compared with the Tm-treated group.

We next observed the expression patterns of AR, EPR, EGF, BTC, HB-EGF, and TGF-α in primary glial cells for 0, 3, 6, 12, and 24 h after the administration of the ER stress antileptic Tm, which induces the unfolded protein production by inhibiting N-linked glycosylation. We found that the expression levels of all EGF family members were increased after 3 h and that there were significant increases in the levels of AR and EPR during the first 6 h; these levels subsequently decreased over time (Fig. 4). However, the expression levels of other family members were maintained at slightly higher levels than those observed in the control cells (S4 Fig.). LDH leakage assays were performed to evaluate the protective effects of recombinant AR and EPR following Tm stimulation, which was used to induce ER Stress in Neuro2a cells. When compared with the control group, we did not observe any statistically significant differences in the LDH leakage gradient concentrations in the recombinant pretreated group, suggesting that the recombinant protein did not induce any toxicity in the Neuro2a cells. However, LDH leakage increased drastically after the addition of Tm. We also found that LDH leakage in the Tm/recombinant pretreated group decreased significantly with increasing concentrations of recombinant AR and EPR recombinant (Fig. 5A and B). Western blotting was then performed to investigate the expression of Transcriptional factor CCAAT/enhancer-binding protein-homologous protein (CHOP) and glucose-regulated protein 78 (GRP78) following Tm stimulation in cells pretreated with recombinant AR and EPR. We found no impact on the expression of CHOP and GRP78 in cells treated with AR and EPR alone. The addition of Tm significantly upregulated the expression of both CHOP and GRP78, and this elevated expression gradually reversed in accordance with increasing concentrations of recombinant AR and EPR (Fig. 6A and B).

## Discussion

Inflammatory reactions, which involve immune responses, are common pathological processes observed in medical clinics. Following LPS stimulation, the levels of 2 pro-inflammatory cytokines were increased in primary glial cells. These increases are considered to be the trigger that initiates an inflammatory reaction. In our study, we screened for EPR and AR expression using a gene chip assay and revealed that the levels of those molecules were drastically higher than those of other EGF family members. A subsequent RT-PCR analysis validated the findings of significant EPR and AR upregulation (Fig. 1A and B). These findings were similar to those observed in a human intestinal epithelial cell line [17]. Shirasawa *et al*. [18] also revealed that EPR may play a critical role in both LPS- and peptidoglycan-induced pro-inflammatory cytokine production in macrophages, whereas Nishimura *et al*. [19] reported that EPR may play an important role in mucosal repair during inflammatory bowel disease. AR has also been reported to induce pro-inflammatory cytokine overexpression in numerous tissues [20]. Moreover, the epidermal growth factor receptor (EGFR) pathway has been reported to interact with inflammatory and immune responses [21]. The EGFR/PI3K/Akt and MAPK pathway is considered to induce pro-inflammatory molecule upregulation and cell proliferation [22]. Based on these studies, it is has been hypothesized that in the brain, AR and EPR are essential for the normal production of pro-inflammatory cytokines involved in the immune response, suggesting that AR and EPR may directly promote inflammatory reactions by inducing pro-inflammatory cytokine production or indirectly stimulate neurogenesis and proliferation by maintaining sufficient numbers of glial cells to attenuate LPS-induced damage.

ER stress, which is induced by the accumulation of unfolded proteins and the disruption of calcium production in the ER, triggers many rescue responses, including the unfolded-protein response (UPR). Excessive or long-term exposure to ER stress may also induce cell apoptosis [23]. In addition, ER stress has been implicated in the pathogenesis of numerous diseases, including neurodegenerative diseases resulting from injury or the apoptosis of neurons and glial cells. GRP78 is an important molecular chaperone located in the ER that acts as a central regulator of multiple ER functions such as participation in the UPR and specific anti-apoptotic actions. CHOP is a known apoptosis-enhancing factor that is present during ER stress. The levels of GRP78 and CHOP expression increase during severe ER stress. In contrast, when ER stress is efficiently inhibited, the levels of GRP78 and CHOP should decrease [24]. Given these findings, GRP78 and CHOP could serve as potential molecular markers of ER stress. We found the increased expression of EPR and AR mRNA following Tm stimulation particularly interesting. Therefore, in our study we used Tm to induce ER stress in primary glial cells and added recombinant EPR and AR in an attempt to inhibit the ER stress response. We found that the expression levels of GRP78 and CHOP protein decreased as the concentrations of added recombinant EPR and AR increased. The downregulation of GRP78 suggests that molecular chaperone recruitment decreased, which correlated with the inhibited production and accumulation of unfolded proteins. Tm induced ER stress and the upregulation of GRP78 functioned as molecular chaperone in cells. The subsequent expression of AR and EPR could attenuate ER stress and UPR, and then suppressing the upregulation of GRP78. Based on the present references, we deduced that the mechanisms underlying the downregulation of GRP78 after expression of AR and EPR involved in EGFR and PI3K/AKT pathway. Intense and persistent ER stress could result in cell apoptosis. It has been reported AR and EPR could inhibit cell apoptosis via the activation of EGFR and PI3K/AKT pathway. Therefore, we made a hypothesis that AR and EPR may inhibit cell apoptosis and attenuate ER stress and UPR by activating AR and EPR and then decrease the expression of GRP78 [25, 26]. The PERK-eIF2α-ATF4 pathway is known to be required for adequate CHOP expression. When severe ER stress occurs, PERK may induce CHOP to promote cell apoptosis by mediating the arrest of cell cycle and protein production [27, 28]. The downregulation of CHOP indicates that the partial restoration of the metabolic pathway in stressed cells suggests the attenuation of apoptosis. Taken together, these findings suggest that EPR and AR function as neuroprotective factors by reducing the need for CHOP and GRP78. The inhibition of CHOP and GRP78, which could be considered the result of a successful UPR, in turn leads to the attenuation of ER stress and subsequent inhibition of apoptosis.

Following hypoxia or cerebral ischemia, affected brain cells undergo a complex series of events that lead to cell death [29]. Cerebral ischemia is a pathophysiological ER stressor and hypoxia is known for inducing ER stress in primary glial cells [15]. Several studies have shown that ischemic injury severely impairs ER function, which in turn triggers the termination of protein translation and apoptosis [30, 31]. These findings suggest that the ER plays an important role in cerebral ischemia. Therefore, reducing ER stress and inducing the UPR survival pathway may comprise a therapeutic method for blocking the pathological process induced by cerebral ischemia [32]. Our data revealed that the upregulation of EGF family members over a short period of time is most likely an important response to the ER stress induced by hypoxic/ischemia in either the tissues or cells. In particular, the expression levels of EPR and particularly AR were remarkably upregulated relative to the control levels in response to hypoxic/ischemic conditions in the cortex and striatum, a finding that is consistent with the results arising from the ER stress-inducing Tm assay presented in Fig. 4. This direct proof associates EPR, AR, and ischemia/hypoxia with ER stress. Moreover, in the hippocampus, which is involved in study and memory, only AR expression exhibited a significant and persistent increase, implying a novel role for AR in protecting the hippocampal neurons. Therefore, it appears likely that EPR and AR are involved in the prevention of cellular death consequent to cerebral ischemia or hypoxia by inhibiting ER stress.

ER stress is not only associated with ischemia and hypoxia but also plays an important role in inflammatory reactions. LPS has been reported as a stressor in primary glial cells and induce ER stress [33]. Recent studies have indicated that inflammatory reactions may induce ER stress. However, the molecular mechanisms underlying these processes remain unclear. ER stress-inducing inflammatory factors have been reported to be mediated by reactive oxygen species (ROS). Therefore, inflammatory reactions may produce ROS that lead to calcium exhaustion and the accumulation of unfolded proteins in the ER, both of which may induce ER stress [34]. In addition, the activation of the UPR signaling pathway during ER stress may also suppress inflammatory reactions by blocking NF-κB activation [35]. Therefore, the upregulation of EPR and AR could partly contribute to the inhibition of ER stress and subsequently the UPR, which triggers inflammatory reactions, thereby protecting tissues and cells from serious damage.

Taken together, the results described in this study suggest that ER stress is a critical component of inflammatory reactions and ischemia/hypoxic progression. EPR and AR most likely exert their neuroprotective effects by inhibiting ER stress, and thus provide a potential therapeutic strategy for attenuating the damage exerted by adverse stimuli in the brain or nervous system.

## Supporting Information

S1 Fig Induction of BTC, EGF, HB-EGF, and TGF-α mRNA expression in primary glial cells following LPS treatment (time course and dose course). (A) Total RNA was isolated from cells exposed to LPS (1 μg/ml) for the indicated time periods and subjected to RT-PCR. (B) Total RNA was isolated from cells exposed to LPS (4 h) at the indicated concentrations and subjected to RT-PCR. Data are presented as the means ± standard errors from 3 separate experiments. *p < 0.05 compared with the control.
(TIF)

S2 Fig Induction of BTC, EGF, HB-EGF, and TGF-α mRNA expression in primary glial cells under hypoxic conditions. Total RNA was isolated from cells exposed to hypoxic conditions for the indicated time periods and subjected to RT-PCR. Data are presented as the means ± standard errors from 3 separate experiments. *p < 0.05 compared with the control.
(TIF)

S3 Fig Time course analysis of BTC, EGF, HB-EGF, and TGF-α mRNA expression in the ischemic cerebral cortex. (A) The injured cortex, (B) striatum, and (C) hippocampus were obtained at various time points (control, 6, 12, 24, and 48 h) following a 30-min hypoxia/ischemia exposure; total RNA was extracted and subjected to RT-PCR. Data are presented as means ± standard errors. Control samples were obtained at 3 h after the sham operation (cont). *p < 0.05 compared with sham-operated mice.
(TIF)

S4 Fig Induction of BTC, EGF, HB-EGF, and TGF-α mRNA expression in primary glial cells following Tm treatment. Total RNA was isolated from cells exposed to Tm (3 μg/ml) for the indicated periods and subjected to RT-PCR. Data are presented as the means ± standard errors from 3 separate experiments. *p < 0.05 compared with the control.
(TIF)

S1 Table Primer sequences used for RT-PCR. (DOC)
