# Supplementary figures and images for: Stress-Induced Neuroprotective Effects of Epiregulin and Amphiregulin
Source: PLoS One. 2015 Feb 12;10(2):e0118280. doi: 10.1371/journal.pone.0118280 (PMC4326420; doi:10.1371/journal.pone.0118280)

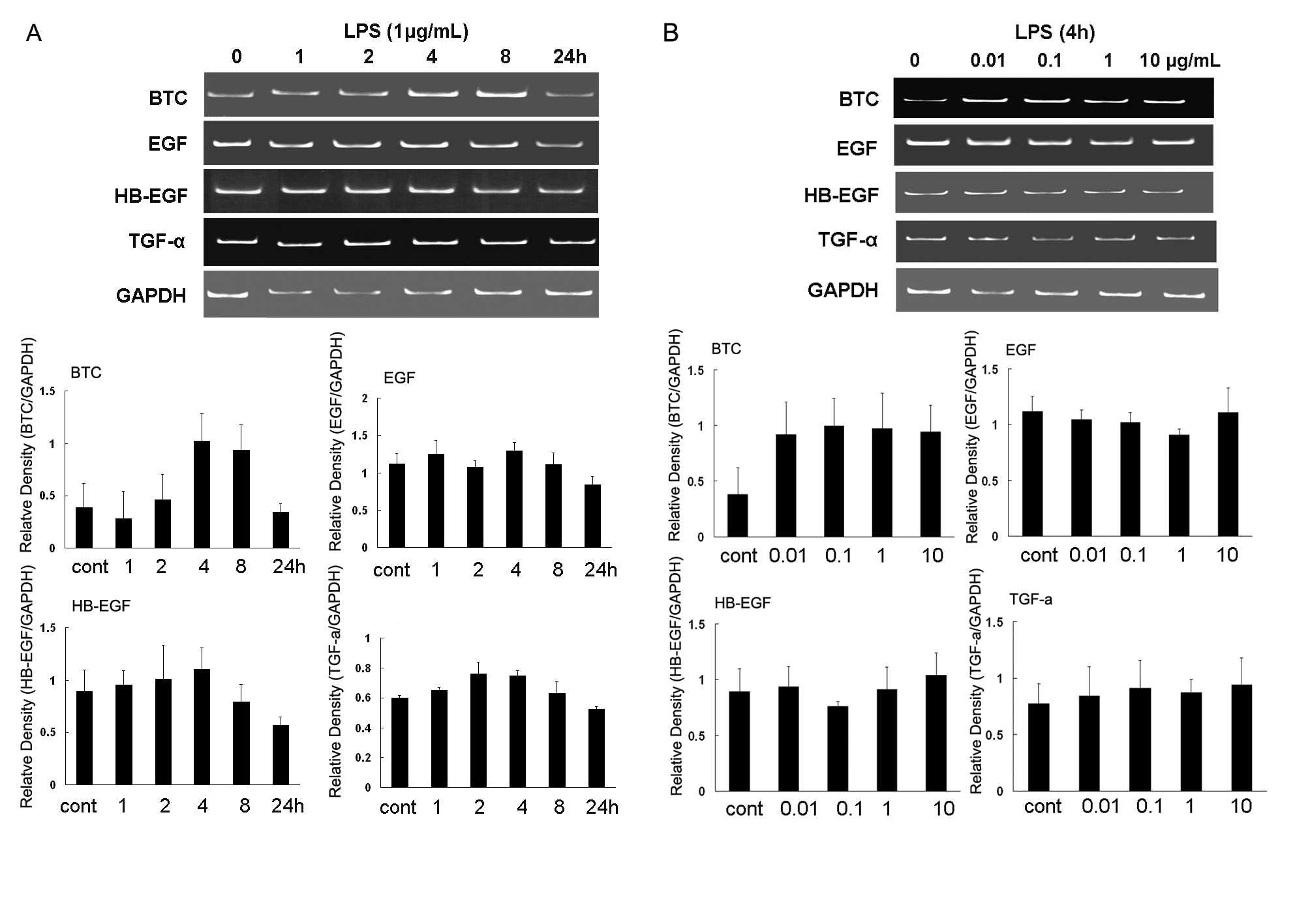

Supplement: S1 Fig — (A) Total RNA was isolated from cells exposed to LPS (1 μg/ml) for the indicated time periods and subjected to RT-PCR. (B) Total RNA was isolated from cells exposed to LPS (4 h) at the indicated concentrations and subjected to RT-PCR. Data are presented as the means ± standard errors from 3 separate experiments. *p < 0.05 compared with the control. (TIF) [file pone.0118280.s001.tif]

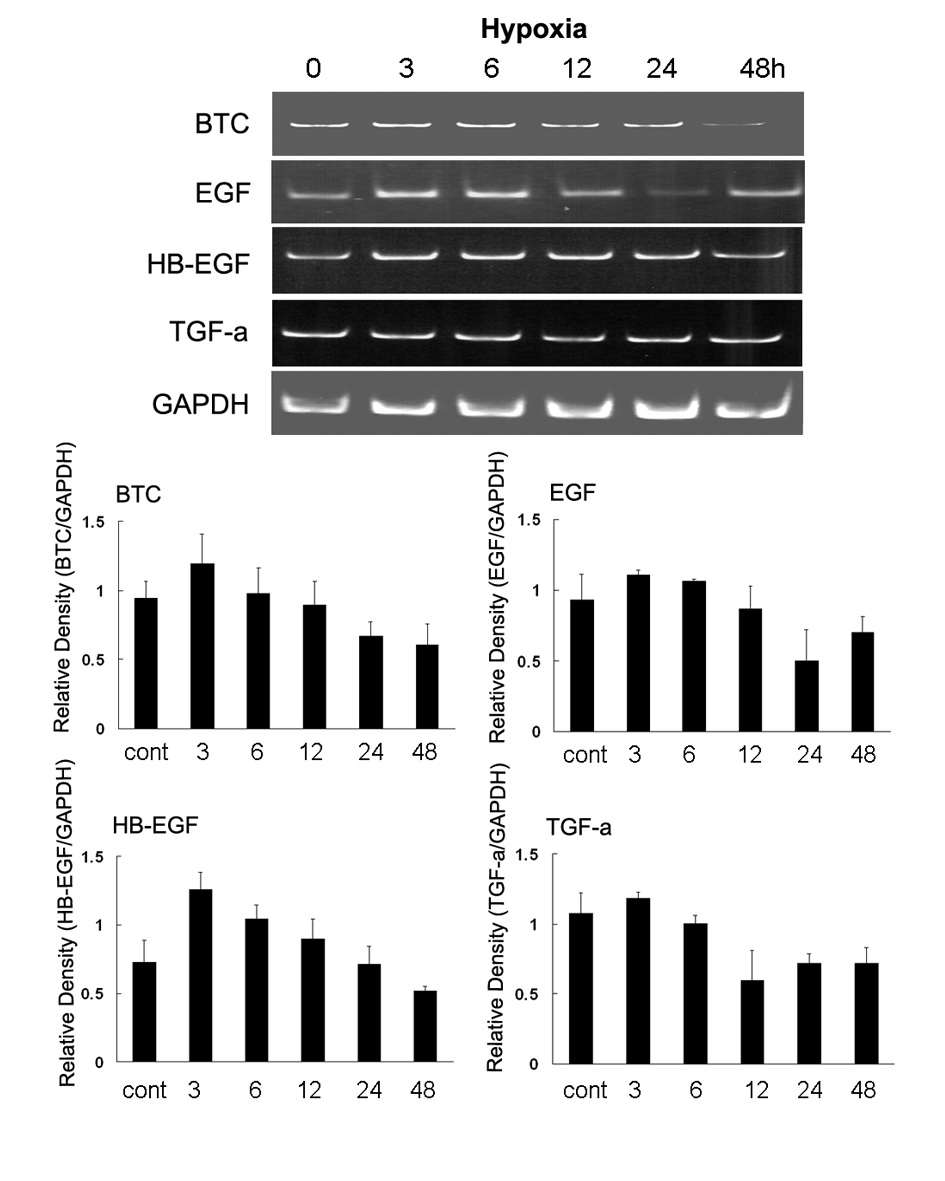

Supplement: S2 Fig — Total RNA was isolated from cells exposed to hypoxic conditions for the indicated time periods and subjected to RT-PCR. Data are presented as the means ± standard errors from 3 separate experiments. *p < 0.05 compared with the control. (TIF) [file pone.0118280.s002.tif]

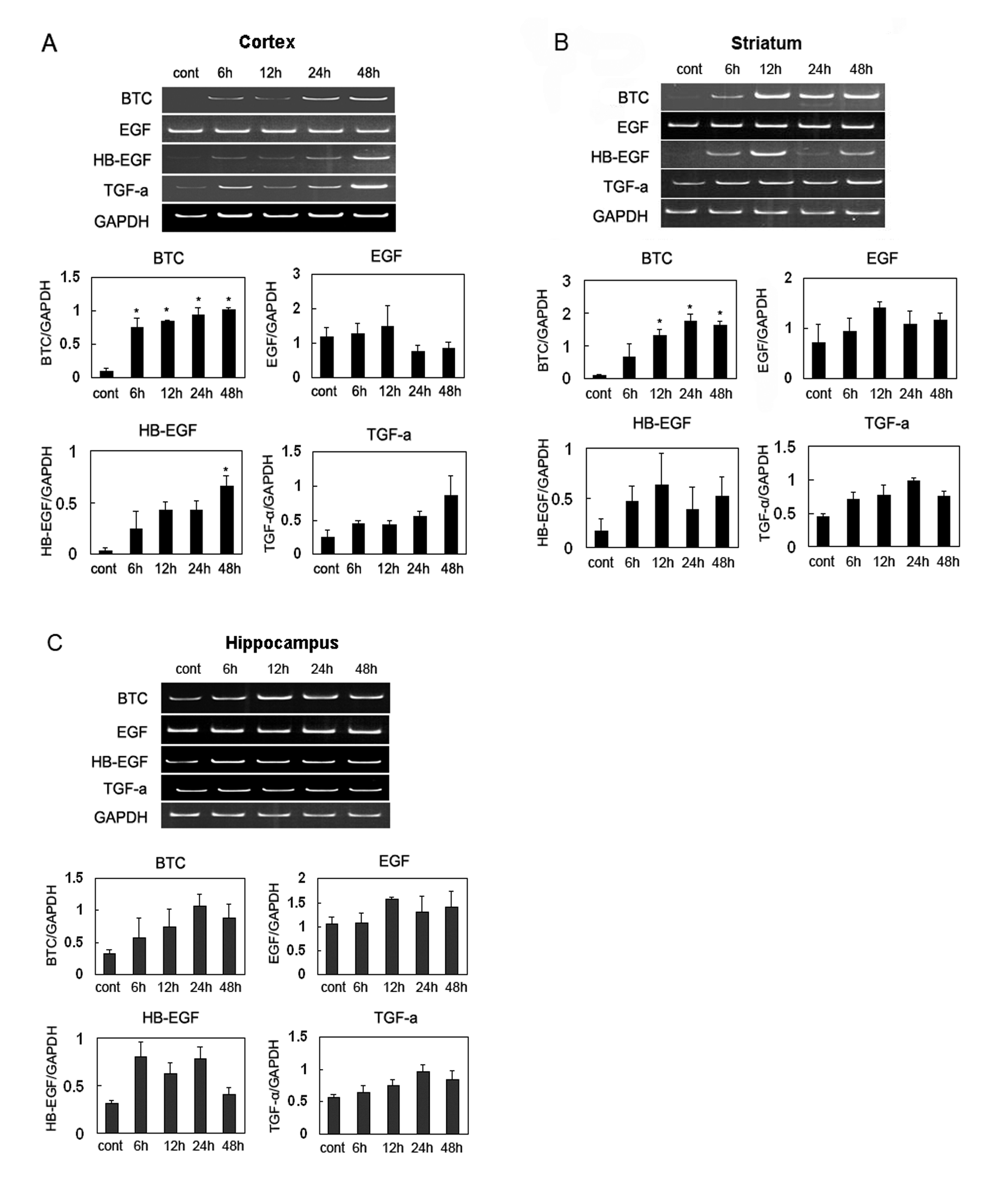

Supplement: S3 Fig — (A) The injured cortex, (B) striatum, and (C) hippocampus were obtained at various time points (control, 6, 12, 24, and 48 h) following a 30-min hypoxia/ischemia exposure; total RNA was extracted and subjected to RT-PCR. Data are presented as means ± standard errors. Control samples were obtained at 3 h after the sham operation (cont). *p < 0.05 compared with sham-operated mice. (TIF) [file pone.0118280.s003.tif]

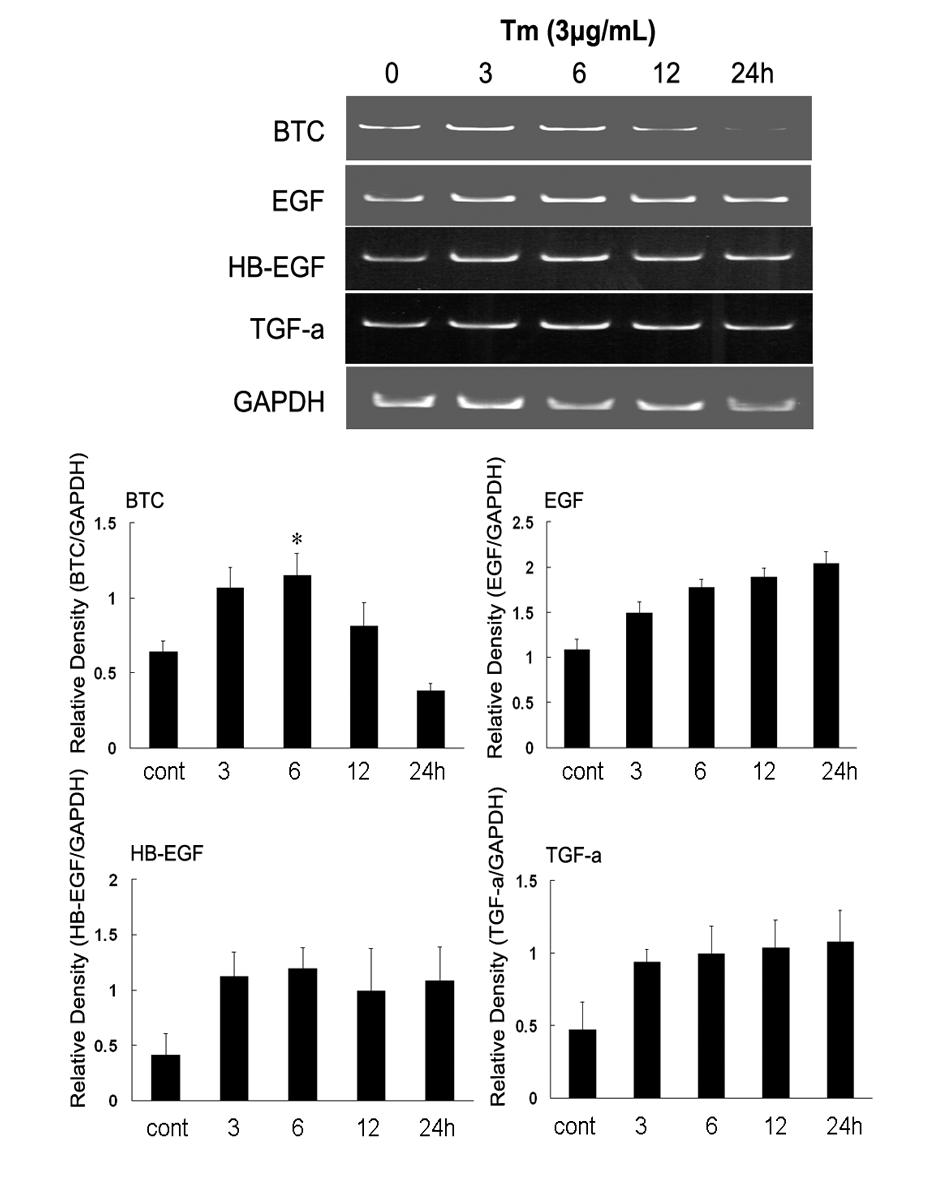

Supplement: S4 Fig — Total RNA was isolated from cells exposed to Tm (3 μg/ml) for the indicated periods and subjected to RT-PCR. Data are presented as the means ± standard errors from 3 separate experiments. *p < 0.05 compared with the control. (TIF) [file pone.0118280.s004.tif]
