# Supplementary material for: Stress-Induced Neuroprotective Effects of Epiregulin and Amphiregulin
Source: PLoS One. 2015 Feb 12;10(2):e0118280. doi: 10.1371/journal.pone.0118280 (PMC4326420; doi:10.1371/journal.pone.0118280)
Supplement: S1 Table — (DOC) [file pone.0118280.s005.doc]

**Table S 1. Primer sequences used for RT-PCR**

|  | Primer | Sequence (5′–3′) | Product (bp) |
| --- | --- | --- | --- |
| EPR | Upstream | ttg ggt cat ctg tta ggt ca | 468 |
|  | Downstream | ccc att gtt att act gtt ta |  |
| AR | Upstream | agt gct gtt gct gct ggt ctt ag | 613 |
|  | Downstream | gat aac gat gcc gat gcc aat a |  |
| EGF | Upstream | ggc ctg gga tgg gaa aat gt | 356 |
|  | Downstream | tgt tga tgc acc tgg acg ag |  |
| BTC | Upstream | cac agc aca gtt gat gga cc | 550 |
|  | Downstream | ccg tta agc aat att ggt ctc |  |
| HB-EGF | Upstream | atg ctg aag ctc ttt ctg g | 589 |
|  | Downstream | cgc cca act tca ctt tct c |  |
| TGF-a | Upstream | gtt ctc agg tcc agc cag tc | 541 |
|  | Downstream | ggt tct ctc ctt cca cca gat |  |
| GAPDH | Upstream | gat gga gca tca tac tga tcc | 361 |
|  | Downstream | aaa ccc atc acc atc ttc cag |  |
